# Testing the Psychometric Properties of an Arabic Version of the Brunel Mood Scale among Physical Education Students

**DOI:** 10.3390/ejihpe13080112

**Published:** 2023-08-18

**Authors:** Hajer Sahli, Faten Sahli, Mouna Saidane, Mahmoud Rebhi, Noomen Guelmami, Khaled Trabelsi, Haitham Jahrami, Achraf Ammar, Peter C. Terry, Makram Zghibi

**Affiliations:** 1 Research Unit, Sportive Performance and Physical Rehabilitation, High Institute of Sports and Physical Education of Kef, University of Jendouba, Kef 7100, Tunisia; sahlihajer2005@yahoo.com (H.S.); ouswamohamed@gmail.com (M.S.); noomen.gualmemi@issepkef.u-jendouba.tn (N.G.); makwiss@yahoo.fr (M.Z.); 2 High Institute of Sport and Physical Education of Sfax, University of Sfax, Sfax 3000, Tunisia; sehli.feten@gmail.com; 3 Research Unit, Physical Activity, Sport and Health, UR18JS01, National Observatory of Sport, Tunis 1003, Tunisia; mahmoudrebhi@issepsf.u-sfax.tn (M.R.); khaled.trabelsi@isseps.usf.tn (K.T.); 4 Research Laboratory, Education, Motricity, Sport and Health, EM2S, LR19JS01, University of Sfax, Sfax 3000, Tunisia; 5 Ministry of Health, Manama 410, Bahrain; hjahrami@health.gov.bh; 6 College of Medicine and Medical Sciences, Arabian Gulf University, Manama 329, Bahrain; 7 Department of Training and Movement Science, Institute of Sport Science, Johannes Gutenberg-University Mainz, 55128 Mainz, Germany; 8 Research Laboratory, Molecular Bases of Human Pathology, LR19ES13, Faculty of Medicine of Sfax, University of Sfax, Sfax 3029, Tunisia; 9 Centre for Health Research, University of Southern Queensland, Toowoomba, QLD 4350, Australia; peter.terry@usq.edu.au

**Keywords:** BRUMS, Arabic-language adaptation, ARAMS, mood, affect, emotion, physical education, students, validity

## Abstract

In our study, we translated and tested the psychometric properties of an Arabic version of the Brunel Mood Scale (BRUMS), referred to as the Arabic Mood Scale (ARAMS), among physical education university students. A total of 681 participants completed the ARAMS in exploratory and confirmatory phases. Exploratory analyses were conducted on data from 253 students between the ages of 19 and 25 years (*M* = 21.14 ± 1.65 years) of whom 132 were women (52.2%) and 121 were men (47.8%). Confirmatory analyses were conducted on data from 428 students between the ages of 19 and 25 years (*M* = 20.93 ± 1.55 years) of whom 203 were women (52.6%) and 225 were men (47.4%). The measurement model of the ARAMS was initially evaluated using exploratory factor analysis (EFA) and was subsequently tested via confirmatory factor analysis (CFA). EFA identified a 24-item, 6-factor structure that aligned with the original BRUMS measurement model, and CFA demonstrated congruence between the two models. Internal consistency of the six subscales exceeded adequacy levels with good Cronbach’s alpha and McDonald’s Omega values respectively for anger (0.811; 0.812), confusion (0.830; 0.830), depression (0.858; 0.859), fatigue (0.823; 0.825), and tension (0.824; 0.825), and an acceptable value for vigor (0.749; 0.748). Findings support the factorial validity and internal consistency of the ARAMS, which appears to be a suitable measure for use in Arabic physical education contexts. Further validation studies are required before the ARAMS is used in other Arabic-language contexts.

## 1. Introduction

Mood is a frequently investigated topic in the field of sports and exercise psychology [1,2] and it has long been proposed that a mood state characterized by a high degree of vigor, combined with lower levels of anger, confusion, depression, fatigue, and tension, is associated with positive mental health [3,4,5,6]. According to McNair et al. [7], moods are transient emotional states, defined for the purposes of our study as “a set of feelings, ephemeral in nature, varying in intensity and duration, and usually involving more than one emotion” [8] p. 16.

Mood profiling is a process in which raw scores on a mood scale are plotted against normative scores to create a graphical profile [9,10]. Mood profiles have been shown to have utility in the prediction of risk of mental health issues. For example, the periodic application of measures such as the Profile of Mood States (POMS) [7] has demonstrated effectiveness in detecting the overtraining syndrome [11,12], which is of great importance in sport contexts. However, some researchers have pointed out that the original 65-item version of the POMS, which has been widely used for mood profiling, is unsuitable in situations where brevity is paramount [13,14], leading to the development of several abbreviated versions. One 37-item abbreviated version was validated specifically for use in cancer patients [13] and another, the 24-item Brunel Mood Scale (BRUMS) [14], was developed initially for use in adolescents and subsequently also validated for use in adult groups [15].

Both the POMS and the BRUMS have been used extensively in the field of sports and exercise psychology to investigate the antecedents, correlates, and behavioral implications of moods, often focusing on the influence of moods on the performance [1,2] and psychological well-being [3,5,16] of athletes and exercisers. For example, meta-analyses conducted by Beedie et al. [1], which summarized 29 studies, and Lochbaum et al. [2], which summarized 25 studies, found that positive moods, characterized by below average scores for tension, depression, anger, fatigue, and confusion, combined with above average scores for vigor, tended to be associated with superior sports performance.

The BRUMS has been used effectively in a wide variety of clinical settings [17,18,19,20,21,22] as well as among healthy individuals [23,24,25,26], becoming a commonly used measure in many cultural contexts. Published translations of the BRUMS have been validated in at least 15 languages, including Afrikaans [27], Bangla [28], Brazilian Portuguese [12], Chinese [29], Czech [30], French [31], Hungarian [32], Italian [32,33], Japanese [34], Lithuanian [35], Malay [36], Persian [37], Serbian [38], Spanish [39], and Turkish [40]. To date, however, there has been no published translation of the BRUMS into Arabic.

There is considerable evidence that physical activity is strongly associated with mood enhancement [41,42,43,44], and tracking mood changes over time has proven to be effective for the purposes of research into the influence of environmental factors on mental health [45,46] and the effects of physical interventions [47,48]. It should be noted that physical education students have not previously been targeted in psychometric studies on mood. Physical education teaching varies from classroom teaching in various ways. In physical education teaching, both theoretical and practical skills are needed [49], requiring those aspiring to enter the profession to gain a theoretical understanding of psychology, sociology, pedagogy, statistics, movement science, biology, and sports, plus practical skills in many sports [50]. Success requires physical performance and psychomotor, emotional, and cognitive skills [51]. In this regard, physical education students are subjected to stressful situations comparable to those of athletes, such as exhaustion and discomfort from multiple physical activities [52]. Physical education students may also experience mental tiredness, circadian rhythm disruption, sleep disturbance, and insomnia from excessive activity levels [53,54,55]. Thus, the assessment of mood among physical education students may be of importance for both diagnostic and intervention purposes. To the best of our knowledge, no psychometric mood research has been carried out on this population. Therefore, the objective of this study was to evaluate the psychometric properties of the ARAMS among physical education students.

## 2. Materials and Methods

### 2.1. Participants

A total of 681 physical education students participated in this study. All participants were enrolled in a bachelor’s degree program in physical education at the High Institute of Physical Education and Sports of Kef at the University of Jendouba, Tunisia. The age of the participants ranged from 19 to 25 years old (*M* = 21.01 ± 1.58 years) with an almost even split between women (*n* = 335, 49.2%) and men (*n* = 346, 51.8%). Of the total sample, 209 (30.7%) were in the first year of the degree program, 282 (41.4%) were in the second year, and 190 (27.9%) were in the third year.

### 2.2. Measure of Mood

The 24-item Brunel Mood Scale [14,15] is a self-report mood inventory of six subscales (tension, depression, anger, vigor, fatigue, and confusion), with four mood descriptors in each subscale. Tension items include “worried” and “anxious”; depression items include “miserable” and “downhearted”; anger items include “angry” and “bad-tempered”; vigor items include “alert” and “energetic”; fatigue items include “tired” and “exhausted”; and confusion items include “muddled” and “uncertain.” Participants rate their responses on a 5-point Likert scale of 0 = not at all, 1 = a little, 2 = moderately, 3 = quite a bit, and 4 = extremely. Evidence to support the factorial and criterion validity of the BRUMS and the internal consistency of the subscales has been provided [14,15] and the measure has been used in a wide variety of research and applied contexts [17,18,19,20,21,22,23,24,25,26].

A cross-cultural translation of the BRUMS into Arabic was conducted in collaboration with two bilingual (Arabic and English) Tunisian psychologists who were aware of the goal of this study. Following the recommended principles for translation–back translation [56,57], the English version of the BRUMS was translated into Arabic by one bilingual psychologist, and then the resultant Arabic version was translated back into English by the other bilingual psychologist. An expert panel of five bilingual clinical psychologists compared the re-translated version to the original form of the scale and carried out necessary modifications to the Arabic item list. Following extensive discussion, the committee reached a consensus on the final item list of the ARAMS, which went forward for testing. The ARAMS is presented in Appendix A.

### 2.3. Procedure

Participants were recruited to join the study via the Institute’s official Facebook page or through direct email. Those who chose to participate completed an online survey that included the ARAMS, which was hosted on Google forms^®^, a cloud-based data management platform. Respondents’ email addresses were recorded, thereby limiting each respondent to providing a single response. Participants were randomly assigned to one of two groups to facilitate both exploratory and confirmatory analyses.

The study protocol received approval from the Ethics Committee of the Higher Institute of Sport and Physical Education of Kef at the University of Jendouba and was undertaken in accordance with the ethical standards of the Declaration of Helsinki in 2013 and its subsequent amendments.

### 2.4. Data Analysis

Data used for the exploratory analysis were collected from 253 students between the ages of 19 and 25 (*M* = 21.14 ± 1.65 years), with 132 women (52.2%) and 121 men (47.8%). Of this subsample, 84 (33.2%) were in the first year of the degree program, 94 (37.2%) were in the second year, and 75 (29.6%) were in the third year. Principal component analysis with Promax rotation was used to explore the factor structure of the ARAMS measure [58]. The reliability of the subscales was examined simultaneously using the Cronbach alpha coefficients (α) and the corrected item-total correlation. For the α coefficient, values above 0.70 were considered as acceptable, above 0.80 as good, and between 0.90 and 0.95 as excellent [59].

Data used for the confirmatory analysis were collected from 428 students between the ages of 19 and 25 (*M* = 20.93 ± 1.55 years), with 225 women (52.6%) and 203 men (47.4%). Of this subsample, 125 (29.2%) were in the first year of the degree program, 188 (43.9%) were in the second year, and 115 (26.9%) were in the third year. The congruence of the ARAMS measurement model with the original measurement model of the BRUMS was tested using confirmatory factor analysis (CFA). In line with recommendations for conducting CFA [60,61,62,63,64,65,66], several fit indices were used in the evaluation. First, the chi-squared (χ^2^) to degrees of freedom ratio was considered, where a ratio of <3 represents an acceptable fit [60]. However, the χ^2^ value is likely to be significant in larger samples with minor model misspecifications, and hence lacked sensitivity as the primary statistic for assessing model fit. We instead gave priority to two incremental fit indices, the non-normed or Tucker–Lewis index (TLI) [61] and the comparative fit index (CFI) [62], both of which adjust for sample size. For the TLI and CFI, values ≥ 0.90 indicate an acceptable fit and values ≥ 0.95 indicate a good fit [63]. We also considered the root mean square error of approximation (RMSEA) [64], which indicates the mean discrepancy between the observed covariances and those implied through the model per degree of freedom, thus also avoiding issues related to larger samples. RMSEA values ≤ 0.05 indicate a good fit and values ≤ 0.08 indicate an acceptable fit [65]. Finally, we used the root mean square residual (SRMR), a measure of the average of the standardized fitted residuals, where a value of ≤0.08 is indicative of an acceptable model [66]. Our sample of 428 participants exceeded the recommended minimum sample size of 10 participants per model parameter for confirmatory factor analysis [66].

## 3. Results

### 3.1. Descriptive Statistics and Distributional Characteristics

Statistical analysis began with the calculation of descriptive statistics and distributional characteristics of the 24 questionnaire items (Table 1).

### 3.2. Exploratory Factor Analysis

**Figure 1:**
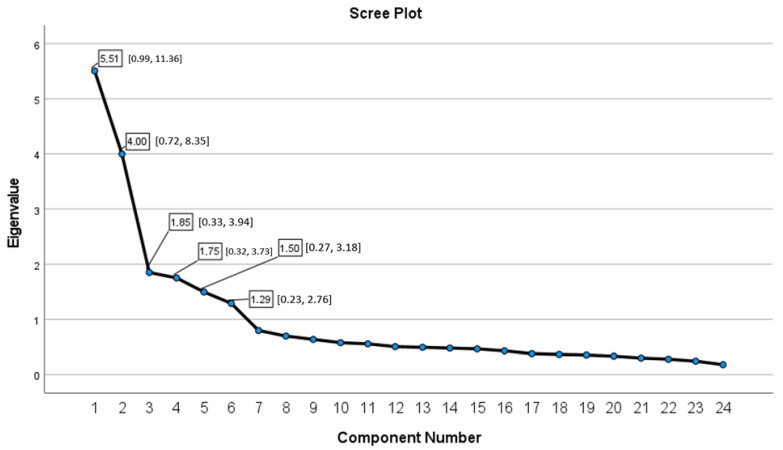
Scree plot of the Arabic Mood Scale.

Results of the principal component analysis, which used the maximum likelihood estimation method with Promax rotation and Kaiser normalization, identified six factors that explained 66.24% of the total variance. The first, second, and third factors explained 22.95% (eigenvalue = 5.51), 16.66% (eigenvalue = 4), and 7.71% (eigenvalue = 1.85) of the variance, respectively, whereas the fourth, fifth, and sixth factors explained 7.30% (eigenvalue = 1.75), 6.24% (eigenvalue = 1.50), and 5.38% (eigenvalue = 1.29), of the variance, respectively. The scree plot for the exploratory solution is shown in Figure 1. The scree plot shows the eigenvalues with the 95% confidence interval of the solution and confirmed the presence of six components (factors) with eigenvalues > 1, with all other possible components adding only minimal additional explained variance, thereby supporting a 6-factor solution as the best interpretation of the data.

The standardized factor loadings indicated a clean solution for the ARAMS that replicated the factor structure of the BRUMS (Table 2). All items loaded onto their hypothesized factor had a factor loading of >0.6, which is considered the acceptable threshold for an established item set [67]. Indeed, 23 of the 24 items (95.23%) had factor loadings of >0.7 (in **bold** type in Table 2), which indicates a good factor loading [68].

### 3.3. Reliability Analysis

The internal consistency of the six factors was assessed using Cronbach alpha coefficients (α). The internal consistency of a factor is judged to be good if the α value is equal to or greater than 0.70. In all instances, α values exceeded 0.70 and for five of the six factors α values exceeded 0.80, indicating that all factors had good internal consistency and that overall, the scale was reliable [66]. Additionally, the McDonald’s Omega values provided for each factor (anger: 0.812, confusion: 0.830, depression: 0.859, fatigue: 0.825, tension: 0.825, and vigor: 0.748) further supported the internal consistency of the scale. Moreover, there were no cases in which an α value for a factor would have increased if an item had been deleted (Table A1, Appendix B).

### 3.4. Confirmatory Factor Analysis

Table 3 presents an overview of the central tendency (*M*), dispersion (*SD*), and distributional shape (kurtosis and skewness) of the 24 items in the confirmatory sample.

**Figure 2:**
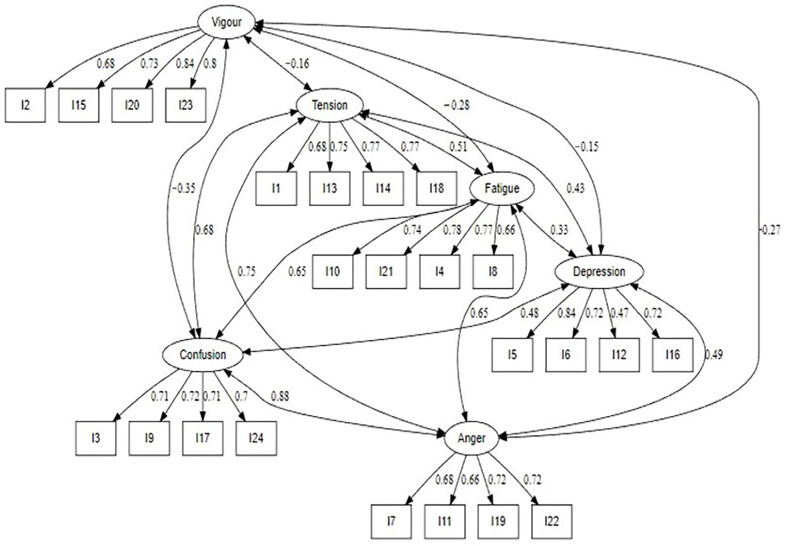
Confirmatory factor analysis (CFA) of the 24-item Arabic Mood Scale.

Figure 2 shows the results of the CFA of the ARAMS. Guidelines [60,63] suggest that a factorial weight > 0.70 is excellent, which was demonstrated by 18 of the 24 items. All items adequately contributed to the hypothesized measurement model, which was shown to be an excellent fit to the data, as confirmed via the fit statistics [TLI = 0.993, CFI = 0.994, RMSEA = 0.014 (90% CI 0.00–0.025), and SRMR = 0.025].

## 4. Discussion

The purpose of the present study was to investigate the reliability and the factorial validity of the Arabic-language version of the BRUMS, referred to as the ARAMS, which was used to assess anger, confusion, depression, fatigue, tension, and vigor, among physical education students. The factor structure of the ARAMS was shown to be identical to that of the original BRUMS. The exploratory factor analysis generated a clear 6-factor structure, while the confirmatory factor analysis showed consistency between the observed model and the theoretical model. Cronbach alpha values showed that the internal consistency of the six subscales was satisfactory in all instances.

These results align closely with several prior cross-cultural validation studies of the BRUMS. For example, Quartiroli et al. [33] used exploratory structural equation modelling techniques to validate the hypothesized measurement model of an Italian translation of the BRUMS among 950 sport participants aged 16 to 63 years. Further, Terry et al. [35] conducted a translation and validation of the BRUMS from English into Lithuanian among 746 general population participants aged 17 to 78 years. Results supported the 24-item, 6-factor measurement model using CFA, and multi-sample analyses supported configural, metric, scalar, and residual invariance across gender groups. These results were replicated using a Bangla version of the BRUMS [28] distributed to 1015 Bangladeshi university students, in which CFA supported the measurement model and showed measurement invariance across participant sex. Recently, a Malay-language version of the BRUMS was tested on a large sample of 4923 Malaysians aged 17 to 75 years [36]. The 24-item, 6-factor measurement model was supported across sex, age, and sport participation using multi-sample CFA. Finally, a cross-cultural validation of the BRUMS using a sample of 1444 English-speaking Singaporeans between the ages of 18 and 65, supported the measurement model, which also showed invariance across sex, age, and sport involvement [69]. In all these studies, the BRUMS subscales showed satisfactory internal consistency.

However, some previous studies have reported a better fit of the measurement model to their data with a reduced set of items. For example, a Farsi translation of the BRUMS showed a reduced 14-item, 6-factor solution that best fit the mood data derived from 405 Iranian university students [37]. Also, in a validation study of a Chinese translation of the BRUMS tested on 2548 participants, Zhang et al. [29] showed that a 23-item, 6-factor measurement model provided an improved fit over the hypothesized model. Similarly, a Spanish validation study of the BRUMS using a sample of 757 respondents aged from 18 to 65 years reported an improved fit of the measurement model when the item pool was reduced in comparison with the original scale [39]. Finally, validation study of a Czech-language version of the BRUMS, conducted using a sample of 246 adolescents, identified a 5-factor measurement model that collapsed the subscales of depression and tension into 1 [30].

This observed equivocality in factor solutions across different languages emphasizes the critical importance of completing a thorough translation process, which ensures that translated items capture cross-cultural nuances in meaning rather than simply capturing literal equivalence. The challenges of translation and cross-cultural validation of health-related questionnaires have been well documented [70,71,72] and may, at least in part, explain why some translations of the BRUMS did not support the proposed measurement model, which is that the translated items did not properly capture the true meaning of the mood descriptors in a new language.

Future cross-cultural validations of questionnaires should consider adopting the multistep translation method advocated recently by Teig et al. [73], which includes two independent forward and back translations, followed by bilingual expert panel scrutiny (both of which were utilized in the present study), but with the addition of Delphi techniques [74] to further establish consensus on translated items. The Delphi technique is an iterative process whereby several individuals across diverse geographical locations and areas of expertise anonymously provide feedback, thus avoiding domination of the consensus process by one or a few experts [75].

Some limitations of the present study are acknowledged. First, all participants were physical education students from a single university in Tunisia and it is not known whether the ARAMS measurement model would be supported equally strongly in other Arabic-speaking groups. Therefore, caution should be exercised when extrapolating the present results to other Arabic populations until further validation studies are completed. Second, it should be noted that participants in our study were experiencing the worst of the COVID-19 restrictions in their country and were also in an examination phase of the studies at the time of data collection. Collectively, the negative effects of COVID-19 on mood [76] coupled with the stress of examinations may have contributed to the mean values of the 24 mood descriptors (see Table 1 and Table 3) which are notably higher than the most recent normative scores reported for the BRUMS [5]. Although these high scores do not influence factor validity, they do preclude the generation of normative data tables for the ARAMS at this stage.

## 5. Conclusions

In our study, we created and evaluated an Arabic-language version of the Brunel Mood Scale, referred to as the Arabic Mood Scale (ARAMS). The hypothesized 24-item, 6-factor structure, and internal consistency of the ARAMS was supported among a sample of 681 physical education students in Tunisia, using exploratory and confirmatory statistical techniques. It is concluded that the ARAMS is a valid and reliable psychometric tool for quantitatively assessing mood states of Arabic-speaking physical education students, although additional validation studies are needed to generalize the use of the ARAMS to other Arabic-speaking populations.

## Figures and Tables

**Table 1:** Descriptive statistics and distributional properties of mood items in the exploratory sample (*n* = 253).

Item	Mood Item	*M*	*SD*	Skewness	Kurtosis
1	Panicky	1.39	0.93	0.26	−0.51
2	Lively	2.29	1.04	0.07	−0.62
3	Confused	1.26	0.89	0.61	0.44
4	Worn out	1.68	0.89	0.19	0.04
5	Depressed	0.93	0.86	0.74	0.32
6	Downhearted	1.00	0.86	0.69	0.31
7	Annoyed	1.45	0.94	0.29	−0.48
8	Exhausted	1.67	0.90	0.27	0.03
9	Mixed-up	1.17	0.91	0.64	0.39
10	Sleepy	1.58	0.93	0.32	−0.13
11	Bitter	1.53	1.10	0.39	−0.45
12	Unhappy	0.95	0.86	0.67	0.18
13	Anxious	1.46	0.92	0.50	0.16
14	Worried	1.40	1.02	0.25	−0.56
15	Energetic	2.25	1.10	0.06	−0.77
16	Miserable	0.91	0.80	0.65	0.22
17	Muddled	1.18	0.93	0.62	0.23
18	Nervous	1.46	0.99	0.39	−0.21
19	Angry	1.50	1.02	0.37	−0.36
20	Active	2.22	1.07	0.04	−0.62
21	Tired	1.63	0.92	0.22	−0.27
22	Bad tempered	1.53	0.97	0.39	−0.15
23	Alert	2.28	0.98	0.21	−0.67
24	Uncertain	1.22	0.94	0.59	0.02

**Table 2:** Standardized factor loadings of the six factors of the Arabic Mood Scale.

Item	Factor					
	Anger	Fatigue	Vigor	Tension	Depression	Confusion
Bad tempered	**0.863**	0.006	−0.349	0.019	0.243	0.107
Angry	**0.789**	−0.007	−0.306	0.035	0.048	0.097
Bitter	**0.812**	0.016	−0.353	0.084	0.258	0.142
Annoyed	**0.812**	−0.078	−0.416	0.026	0.272	−0.051
Worn out	0.001	**0.852**	0.038	0.363	0.229	0.280
Sleepy	−0.011	**0.802**	0.045	0.293	0.164	0.173
Tired	−0.133	**0.806**	0.032	0.305	0.148	0.262
Exhausted	0.108	**0.796**	−0.091	0.368	0.246	0.257
Energetic	−0.285	−0.016	**0.838**	0.002	−0.263	−0.127
Alert	−0.381	−0.048	**0.828**	−0.065	−0.245	−0.016
Lively	−0.396	0.045	**0.797**	−0.058	−0.031	0.059
Active	−0.360	0.028	**0.814**	0.028	−0.149	0.036
Anxious	0.136	0.404	−0.041	**0.824**	0.293	0.282
Worried	0.096	0.365	−0.103	**0.835**	0.398	0.344
Panicky	−0.108	0.254	0.166	**0.780**	0.333	0.178
Nervous	0.067	0.360	−0.088	**0.823**	0.398	0.390
Miserable	0.280	0.204	−0.202	0.384	**0.836**	0.195
Downhearted	0.065	0.143	−0.100	0.255	**0.758**	0.171
Depressed	0.237	0.295	−0.245	0.330	**0.790**	0.310
Unhappy	0.257	0.150	−0.135	0.409	**0.731**	0.186
Uncertain	0.120	0.319	0.053	0.315	0.295	**0.817**
Muddled	0.147	0.228	−0.061	0.368	0.234	**0.793**
Mixed-up	−0.048	0.049	−0.053	0.165	0.053	**0.661**
Confused	0.123	0.326	0.031	0.194	0.244	**0.701**

**Table 3:** Distributional properties of mood items in the confirmatory sample (*n* = 428).

Item	*M*	*SD*	Skewness	Kurtosis
Panicky	2.42	0.81	−0.23	−0.34
Lively	2.44	0.82	−0.19	−0.22
Confused	2.43	0.80	0.01	−0.08
Worn out	2.44	0.81	−0.27	−0.17
Depressed	2.35	0.83	−0.13	−0.14
Downhearted	2.41	0.83	−0.16	−0.19
Annoyed	2.32	0.79	−0.02	−0.14
Exhausted	2.39	0.88	−0.22	−0.37
Mixed-up	1.75	0.83	−0.17	−0.45
Sleepy	1.78	0.88	−0.03	−0.48
Bitter	1.73	0.89	0.02	−0.44
Unhappy	1.74	0.91	−0.12	−0.64
Anxious	2.40	0.84	−0.05	−0.65
Worried	2.44	0.84	−0.26	−0.45
Energetic	2.46	0.86	−0.24	−0.49
Miserable	2.48	0.87	−0.34	−0.60
Muddled	2.29	0.84	−0.08	−0.58
Nervous	2.31	0.80	−0.22	−0.39
Angry	2.29	0.80	−0.21	−0.58
Active	2.24	0.81	0.03	−0.58
Tired	2.43	0.79	−0.27	−0.28
Bad tempered	2.38	0.83	−0.05	−0.62
Alert	2.41	0.85	−0.14	−0.29
Uncertain	2.35	0.81	−0.27	−0.39

## Data Availability

Data are available from the corresponding author.

## Appendix A. Arabic Mood Scale (ARAMS) and Scoring Instructions

هذا المقياس مكون من 24 عنصرًا عبارة عن جرد تقرير ذاتي للمزاج من ستة مقاييس فرعية (التوتر ، والاكتئاب ، والغضب ، والحيوية ، والتعب ، والارتباك) ، مع أربعة توصيفات للمزاج في كل مقياس فرعي. على مقياس ليكرت المكون من 5 نقاط من 0 = لا على الإطلاق ، 1 = قليلًا ، 2 = متوسط ، 3 = كافٍ ، 4 = للغاية.
1. الحيوية (2،15،20،23) 2. التوتر (1،13،14،18) 3. التعب (10،21،4،8) 4. الاكتئاب (5،6،12،16) 5. الارتباك (3،9،17،24) 6. الغضب (7،11،19،22)
**مقياس** **آرامس**
التعليمات: قيّم ما تشعر به بوضع علامة (X) في الخانة المناسبة.

للغاية	كافٍ	متوسط	قليلاً	لا على الإطلاق		
					مضطرب	1
					حي	2
					حائر	3
					منهك	4
					محبَط	5
					مكتئب	6
					متضايق	7
					مرهق	8
					مختلط	9
					نعسان	10
					على مرارة	11
					غير سعيد	12
					قلق	13
					مهموم	14
					نشيط	15
					بائس	16
					مشوش	17
					متوتر	18
					غاضب	19
					نشيط	20
					متعب	21
					سيء المزاج	22
					على انتباه	23
					غير متأكد	24

## Appendix B

**Table A1:** Reliability (α) coefficients of the Arabic Mood Scale.

Factor	Item	Item-Total r	Alpha (α) If Item Deleted	Alpha (α)	McDonald’s Omega
Anger	I7	0.663	0.746	0.811	0.812
	I11	0.615	0.770		
	I19	0.581	0.785		
	I22	0.657	0.749		
Confusion	I3	0.663	0.783	0.830	0.830
	I9	0.640	0.794		
	I17	0.663	0.784		
	I24	0.668	0.782		
Depression	I5	0.701	0.820	0.858	0.859
	I6	0.670	0.832		
	I12	0.687	0.826		
	I16	0.753	0.798		
Fatigue	I4	0.684	0.760	0.823	0.825
	I8	0.594	0.800		
	I10	0.625	0.788		
	I21	0.686	0.759		
Tension	I1	0.610	0.796	0.824	0.825
	I13	0.633	0.787		
	I14	0.669	0.770		
	I18	0.692	0.762		
Vigor	I2	0.554	0.686	0.749	0.748
	I15	0.503	0.714		
	I20	0.541	0.693		
	I23	0.578	0.672		
